# Drug Overdose Deaths Involving Cocaine and Psychostimulants with Abuse Potential — United States, 2003–2017

**DOI:** 10.15585/mmwr.mm6817a3

**Published:** 2019-05-03

**Authors:** Mbabazi Kariisa, Lawrence Scholl, Nana Wilson, Puja Seth, Brooke Hoots

**Affiliations:** 1 Division of Unintentional Injury Prevention, National Center for Injury Prevention and Control, CDC.

In 2016, a total of 63,632 persons died from drug overdoses in the United States (*1*). Drug overdose deaths involving cocaine, psychostimulants with abuse potential (psychostimulants), or both substances combined increased 42.4% from 12,122 in 2015 to 17,258 in 2016.* Psychostimulants with abuse potential include drugs such as methamphetamine, 3,4-methylenedioxy-methamphetamine (MDMA), dextroamphetamine, levoamphetamine, methylphenidate (Ritalin), and caffeine. From 2015 to 2016, cocaine-involved and psychostimulant-involved death rates increased 52.4% and 33.3%, respectively (*1*). A total of 70,237 persons died from drug overdoses in the United States in 2017; approximately two thirds of these deaths involved an opioid (*2*). CDC analyzed 2016–2017 changes in age-adjusted death rates involving cocaine and psychostimulants by demographic characteristics, urbanization levels, U.S. Census region, 34 states, and the District of Columbia (DC). CDC also examined trends in age-adjusted cocaine-involved and psychostimulant-involved death rates from 2003 to 2017 overall, as well as with and without co-involvement of opioids. Among all 2017 drug overdose deaths, 13,942 (19.8%) involved cocaine, and 10,333 (14.7%) involved psychostimulants. Death rates increased from 2016 to 2017 for both drug categories across demographic characteristics, urbanization levels, Census regions, and states. In 2017, opioids were involved in 72.7% and 50.4% of cocaine-involved and psychostimulant-involved overdoses, respectively, and the data suggest that increases in cocaine-involved overdose deaths from 2012 to 2017 were driven primarily by synthetic opioids. Conversely, increases in psychostimulant-involved deaths from 2010 to 2017 occurred largely independent of opioids, with increased co-involvement of synthetic opioids in recent years. Provisional data from 2018 indicate that deaths involving cocaine and psychostimulants are continuing to increase.^†^ Increases in stimulant-involved deaths are part of a growing polysubstance landscape. Increased surveillance and evidence-based multisectoral prevention and response strategies are needed to address deaths involving cocaine and psychostimulants and opioids. Enhancing linkage to care, building state and local capacity, and public health/public safety collaborations are critical components of prevention efforts.

Drug overdose deaths were identified in the National Vital Statistics System multiple cause-of-death mortality files,^§^ using *International Classification of Diseases, Tenth Revision* (ICD-10) underlying cause-of-death codes X40–X44 (unintentional), X60–X64 (suicide), X85 (homicide), or Y10–Y14 (undetermined intent). Among deaths with drug overdose as the underlying cause, the type of drug is indicated by the following ICD-10 multiple cause-of-death codes: cocaine (T40.5); psychostimulants with abuse potential (T43.6); opioids (T40.0–T40.4, and T40.6)^¶^; and synthetic opioids other than methadone (T40.4). Some deaths involved more than one type of drug; these deaths were included in the rates for each drug category. Thus, categories were not mutually exclusive.**

Age-adjusted death rates^††^ were examined for the period 2016– 2017 for cocaine and psychostimulants. Death rates were stratified by age group, sex, race/ethnicity, urbanization level,^§§^ U.S. Census region,^¶¶^ and state. State-level analyses were conducted for 34 states and DC, all of which had adequate drug-specificity data recorded on death certificates for 2016 and 2017.*** Analyses comparing changes in death rates from 2016 to 2017 used z-tests when deaths were ≥100 and nonoverlapping confidence intervals based on a gamma distribution when deaths were <100.^†††^ Trends in age-adjusted cocaine-involved and psychostimulant-involved death rates from 2003 to 2017 were analyzed overall, and with and without any opioids and synthetic opioids, using Joinpoint regression.^§§§^ Changes presented represent statistically significant findings unless otherwise specified.

In 2017, among 70,237 drug overdose deaths that occurred in the United States, 13,942 (19.8%) involved cocaine, representing a 34.4% increase from 2016 (Table). Nearly three fourths (72.7%) of cocaine-involved deaths in 2017 also involved opioids. Cocaine-involved death rates increased among both sexes and among persons aged ≥15 years, non-Hispanic whites (whites), non-Hispanic blacks (blacks), and Hispanics. The largest relative rate change occurred among females aged 15–24 years (40.0%), and the largest absolute rate change was among males aged 25–44 and 45–64 years (increase of 2.7 per 100,000). Among racial/ethnic groups, the highest rate of cocaine-involved deaths in 2017 occurred in blacks (8.3 per 100,000), who also experienced the largest relative rate change (36.1%) compared with 2016. By urban-rural status, counties in medium metro areas experienced the largest absolute rate increase (1.3 per 100,000) in 2017, whereas the largest relative rate increase occurred in micropolitan counties (57.9%). The Midwest Census region had the largest relative rate increase (43.6%), whereas the highest 2017 rate was in the Northeast (7.0 per 100,000). Death rates involving cocaine increased in 15 states, with the largest relative increases in Wisconsin (84.6%) and Maryland (72.0%), and the largest absolute rate increases in Ohio (3.9) and Maryland (3.6). In 2017, the highest death rates were in DC (17.6) and Ohio (14.0).

**TABLE:** Number and age-adjusted rate of drug overdose deaths* involving cocaine^†^ and psychostimulants with abuse potential,^§^^,^^¶^ by opioid involvement,** sex, age group, race and Hispanic origin,^††^ U.S. Census region, urbanization level,^§§^ and selected states^¶¶^ — United States, 2016 and 2017

Decedent characteristic	Involving cocaine				Involving psychostimulants with abuse potential			
	2016	2017	Change from 2016 to 2017***		2016	2017	Change from 2016 to 2017***	
	No. (Rate)	No. (Rate)	Absolute rate change	% Change in rate	No. (Rate)	No. (Rate)	Absolute rate change	% Change in rate
**Overall**	**10,375 (3.2)**	**13,942 (4.3)**	**1.1^†††^**	**34.4^†††^**	**7,542 (2.4)**	**10,333 (3.2)**	**0.8^†††^**	**33.3^†††^**
With any opioid**	7,263 (2.3)	10,131 (3.2)	0.9^†††^	39.1^†††^	3,416 (1.1)	5,203 (1.7)	0.6^†††^	54.5^†††^
**Sex**								
Male	7,493 (4.7)	10,021 (6.2)	1.5^†††^	31.9^†††^	5,348 (3.4)	7,240 (4.5)	1.1^†††^	32.4^†††^
Female	2,882 (1.8)	3,921 (2.5)	0.7^†††^	38.9^†††^	2,194 (1.4)	3,093 (1.9)	0.5^†††^	35.7^†††^
**Age group (yrs)**								
0–14	^§§§^	^§§§^	^§§§^	^§§§^	11^§§§^	^§§§^	^§§§^	^§§§^
15–24	757 (1.7)	924 (2.1)	0.4^†††^	23.5^†††^	571 (1.3)	780 (1.8)	0.5^†††^	38.5^†††^
25–34	2,525 (5.7)	3,463 (7.6)	1.9^†††^	33.3^†††^	1,762 (3.9)	2,593 (5.7)	1.8^†††^	46.2^†††^
35–44	2,431 (6.0)	3,282 (8.0)	2.0^†††^	33.3^†††^	1,831 (4.5)	2,548 (6.2)	1.7^†††^	37.8^†††^
45–54	2,629 (6.1)	3,497 (8.3)	2.2^†††^	36.1^†††^	1,914 (4.5)	2,477 (5.8)	1.3^†††^	28.9^†††^
55–64	1,721 (4.2)	2,335 (5.6)	1.4^†††^	33.3^†††^	1,244 (3.0)	1,648 (3.9)	0.9^†††^	30.0^†††^
≥65	303 (0.6)	432 (0.8)	0.2^†††^	33.3^†††^	206 (0.4)	278 (0.5)	0.1^†††^	25.0^†††^
**Sex/Age group (yrs)**								
**Male**								
15–24	553 (2.5)	633 (2.9)	0.4^†††^	16.0^†††^	388 (1.7)	499 (2.3)	0.6^†††^	35.3^†††^
25–44	3,569 (8.3)	4,784 (11.0)	2.7^†††^	32.5^†††^	2,536 (5.9)	3,551 (8.2)	2.3^†††^	39.0^†††^
45–64	3,108 (7.6)	4,229 (10.3)	2.7^†††^	35.5^†††^	2,251 (5.5)	2,955 (7.2)	1.7^†††^	30.9^†††^
**Female**								
15–24	204 (1.0)	291 (1.4)	0.4^†††^	40.0^†††^	183 (0.9)	281 (1.3)	0.4^†††^	44.4^†††^
25–44	1,387 (3.3)	1,961 (4.6)	1.3^†††^	39.4^†††^	1,057 (2.5)	1,590 (3.7)	1.2^†††^	48.0^†††^
45–64	1,242 (2.9)	1,603 (3.7)	0.8^†††^	27.6^†††^	907 (2.1)	1,170 (2.7)	0.6^†††^	28.6^†††^
**Race and Hispanic origin^††^**								
White, non-Hispanic	6,443 (3.4)	8,614 (4.6)	1.2^†††^	35.3^†††^	5,777 (3.0)	7,995 (4.2)	1.2^†††^	40.0^†††^
Black, non-Hispanic	2,599 (6.1)	3,554 (8.3)	2.2^†††^	36.1^†††^	477 (1.2)	663 (1.6)	0.4^†††^	33.3^†††^
Hispanic	1,097 (2.0)	1,438 (2.5)	0.5^†††^	25.0^†††^	846 (1.5)	1,125 (2.0)	0.5^†††^	33.3^†††^
American Indian/Alaska Native, non-Hispanic	56 (2.1)	65 (2.4)	0.3	14.3	181 (6.9)	222 (8.5)	1.6^†††^	23.2^†††^
Asian/Pacific Islander, non-Hispanic	85 (0.4)	129 (0.6)	0.2	50.0	171 (0.8)	218 (1.0)	0.2^†††^	25.0^†††^
**U.S. Census region of residence**								
Northeast	2,957 (5.3)	3,860 (7.0)	1.7^†††^	32.1^†††^	431 (0.8)	648 (1.2)	0.4^†††^	50.0^†††^
Midwest	2,575 (3.9)	3,711 (5.6)	1.7^†††^	43.6^†††^	1,176 (1.9)	1,959 (3.1)	1.2^†††^	63.2^†††^`
South	4,005 (3.3)	5,365 (4.4)	1.1^†††^	33.3^†††^	2,483 (2.1)	3,508 (3.0)	0.9^†††^	42.9^†††^
West	838 (1.1)	1,006 (1.3)	0.2^†††^	18.2^†††^	3,452 (4.4)	4,218 (5.3)	0.9^†††^	20.5^†††^
**County urbanization level** ^§§^								
Large central metro	4,301 (4.2)	5,513 (5.3)	1.1^†††^	26.2^†††^	2,561 (2.5)	3,178 (3.0)	0.5^†††^	20.0^†††^
Large fringe metro	2,734 (3.5)	3,701 (4.7)	1.2^†††^	34.3^†††^	1,235 (1.6)	1,843 (2.3)	0.7^†††^	43.8^†††^
Medium metro	2,082 (3.2)	2,945 (4.5)	1.3^†††^	40.6^†††^	1,821 (2.8)	2,672 (4.1)	1.3^†††^	46.4^†††^
Small metro	569 (2.1)	777 (2.9)	0.8^†††^	38.1^†††^	698 (2.6)	972 (3.6)	1.0^†††^	38.5^†††^
Micropolitan (non-metro)	474 (1.9)	740 (3.0)	1.1^†††^	57.9^†††^	745 (3.0)	994 (4.0)	1.0^†††^	33.3^†††^
Non-core (non-metro)	215 (1.3)	266 (1.6)	0.3^†††^	23.1^†††^	482 (2.9)	674 (4.1)	1.2^†††^	41.4^†††^
**States with very good to excellent reporting**^¶¶^ **(n = 27)**								
Alaska	15^§§§^	17^§§§^	^§§§^	^§§§^	49 (6.3)	66 (9.1)	2.8	44.4
Connecticut	237 (6.9)	284 (8.4)	1.5^†††^	21.7^†††^	25 (0.7)	39 (1.2)	0.5	71.4
District of Columbia	89 (13.5)	122 (17.6)	4.1	30.4	^§§§^	^§§§^	^§§§^	^§§§^
Georgia	209 (2.0)	258 (2.4)	0.4	20.0	243 (2.4)	364 (3.6)	1.2^†††^	50.0^†††^
Hawaii	^§§§^	10^§§§^	^§§§^	^§§§^	102 (6.8)	106 (7.4)	0.6	8.8
Illinois	507 (4.0)	743 (5.7)	1.7^†††^	42.5^†††^	112 (0.9)	171 (1.4)	0.5^†††^	55.6^†††^
Iowa	15^§§§^	19^§§§^	^§§§^	^§§§^	80 (2.7)	93 (3.3)	0.6	22.2
Maine	61 (5.0)	94 (7.7)	2.7	54.0	28 (2.3)	44 (3.8)	1.5	65.2
Maryland	314 (5.0)	532 (8.6)	3.6^†††^	72.0^†††^	43 (0.8)	65 (1.2)	0.4	50.0
Massachusetts	567 (8.5)	687 (10.1)	1.6^†††^	18.8^†††^	45 (0.7)	64 (1.0)	0.3	42.9
Nevada	37 (1.2)	50 (1.6)	0.4	33.3	228 (7.5)	257 (8.3)	0.8	10.7
New Hampshire	61 (5.0)	51 (3.9)	−1.1	−22.0	13^§§§^	26 (2.3)	^§§§^	^§§§^
New Mexico	58 (3.0)	57 (2.9)	−0.1	−3.3	135 (7.1)	158 (8.2)	1.1	15.5
New York	991 (4.9)	1,306 (6.5)	1.6^†††^	32.7^†††^	150 (0.8)	191 (1.0)	0.2^†††^	25.0^†††^
North Carolina	500 (5.1)	708 (7.2)	2.1^†††^	41.2^†††^	115 (1.2)	176 (1.8)	0.6^†††^	50.0^†††^
Ohio	1,124 (10.1)	1,556 (14.0)	3.9^†††^	38.6^†††^	243 (2.3)	556 (5.3)	3.0^†††^	130.4^†††^
Oklahoma	31 (0.8)	45 (1.1)	0.3	37.5	263 (7.1)	275 (7.2)	0.1	1.4
Oregon	26 (0.7)	39 (0.9)	0.2	28.6	150 (3.6)	170 (4.0)	0.4	11.1
Rhode Island	112 (10.7)	111 (11.2)	0.5	4.7	10^§§§^	12^§§§^	^§§§^	^§§§^
South Carolina	143 (3.0)	234 (4.7)	1.7^†††^	56.7^†††^	125 (2.7)	189 (4.0)	1.3^†††^	48.1^†††^
Tennessee	249 (3.8)	306 (4.6)	0.8^†††^	21.1^†††^	186 (2.9)	320 (5.0)	2.1^†††^	72.4^†††^
Utah	48 (1.7)	47 (1.5)	−0.2	−11.8	143 (5.1)	198 (6.8)	1.7^†††^	33.3^†††^
Vermont	21 (4.0)	38 (6.9)	2.9	72.5	^§§§^	^§§§^	^§§§^	^§§§^
Virginia	254 (3.0)	351 (4.1)	1.1^†††^	36.7^†††^	76 (0.9)	113 (1.4)	0.5	55.6
Washington	90 (1.2)	111 (1.4)	0.2	16.7	326 (4.4)	392 (5.2)	0.8^†††^	18.2^†††^
West Virginia	143 (8.5)	191 (11.6)	3.1^†††^	36.5^†††^	117 (7.0)	221 (13.6)	6.6^†††^	94.3^†††^
Wisconsin	147 (2.6)	265 (4.8)	2.2^†††^	84.6^†††^	76 (1.4)	128 (2.3)	0.9^†††^	64.3^†††^
**States with good reporting**^¶¶^ **(n = 8)**								
Arizona	82 (1.2)	136 (2.0)	0.8^†††^	66.7^†††^	454 (6.7)	572 (8.5)	1.8^†††^	26.9^†††^
California	366 (0.9)	433 (1.0)	0.1	11.1	1,579 (3.8)	1,916 (4.6)	0.8^†††^	21.1^†††^
Colorado	106 (1.9)	96 (1.7)	−0.2	−10.5	200 (3.6)	301 (5.2)	1.6^†††^	44.4^†††^
Kentucky	145 (3.5)	185 (4.3)	0.8	22.9	192 (4.7)	330 (8.0)	3.3^†††^	70.2^†††^
Michigan	500 (5.3)	643 (6.7)	1.4^†††^	26.4^†††^	88 (0.9)	145 (1.6)	0.7^†††^	77.8^†††^
Minnesota	43 (0.8)	68 (1.3)	0.5	62.5	140 (2.6)	161 (2.9)	0.3	11.5
Missouri	103 (1.8)	132 (2.2)	0.4	22.2	185 (3.3)	248 (4.3)	1.0^†††^	30.3^†††^
Texas	584 (2.1)	694 (2.4)	0.3^†††^	14.3^†††^	577 (2.1)	653 (2.3)	0.2	9.5

**Source:** National Vital Statistics System, Mortality File. https://wonder.cdc.gov/.

* Deaths are classified using *the International Classification of Diseases, Tenth Revision* (ICD–10). Drug overdose deaths are identified using underlying cause-of-death codes X40–X44, X60–X64, X85, and Y10–Y14. Rates are age-adjusted using the direct method and the 2000 U.S. standard population, except for age-specific crude rates. All rates are per 100,000 population.

^†^ Drug overdose deaths, as defined, that have cocaine (T40.5) as a contributing cause.

^§^ Drug overdose deaths, as defined, that have psychostimulants with abuse potential (T43.6) as a contributing cause.

^¶^ Categories of deaths are not exclusive because deaths might involve more than one drug. Summing of categories will result in more than the total number of deaths in a year.

** Drug overdose deaths, as defined, that have any opioid (T40.0–T40.4, and T40.6).

^††^ Data for Hispanic origin should be interpreted with caution; studies comparing Hispanic origin on death certificates and on census surveys have shown inconsistent reporting on Hispanic ethnicity. Potential race misclassification might lead to underestimates for certain categories, primarily American Indian/Alaska Native non-Hispanic and Asian/Pacific Islander non-Hispanic decedents. https://www.cdc.gov/nchs/data/series/sr_02/sr02_172.pdf.

^§§^ By 2013 urbanization classification https://www.cdc.gov/nchs/data_access/urban_rural.htm.

^¶¶^ Analyses were limited to states meeting the following criteria: For states with very good to excellent reporting, ≥90% of drug overdose deaths mention at least one specific drug in 2016, with the change in drug overdose deaths mentions of at least one specific drug differing by <10 percentage points between 2016 and 2017. States with good reporting had 80% to <90% of drug overdose deaths mention of at least one specific drug in 2016, with the change in the percentage of drug overdose deaths mentioning at least one specific drug differing by <10 percentage points between 2016 and 2017. States included also were required to have stable rate estimates, based on ≥20 deaths, in at least one drug category (i.e., cocaine and psychostimulants with abuse potential) in both 2016 and 2017.

*** Absolute rate change is the difference between 2016 and 2017 rates. Percentage change (i.e., relative change) is the absolute rate change divided by the 2016 rate, multiplied by 100. Nonoverlapping confidence intervals based on the gamma method were used if the number of deaths was <100 in 2016 or 2017, and z-tests were used if the number of deaths was ≥100 in both 2016 and 2017. Note that the method of comparing confidence intervals is a conservative method for statistical significance; caution should be observed when interpreting a nonsignificant difference when the lower and upper limits being compared overlap only slightly. Confidence intervals for 2016 and 2017 rates of cocaine-involved deaths for Asian/Pacific Islanders overlapped only slightly: (0.35–0.54), (0.53–0.76) Confidence intervals of 2016 and 2017 rates of deaths involving psychostimulants with abuse potential for Virginia overlapped only slightly: (0.71–1.13), (1.10–1.60).

^†††^ Statistically significant (p-value <0.05).

^§§§^ Data with <10 deaths are not reported. Rates based on <20 deaths are not considered reliable and not reported.

During 2003–2017, rates for all cocaine-involved deaths peaked initially in 2006, decreased during 2006–2012, and increased again during 2012–2017. Rates of overdose deaths involving cocaine and any opioid increased from 2013 to 2017, and those involving cocaine and synthetic opioids increased from 2012 to 2017 (Figure 1). Cocaine-involved death rates without any opioid decreased from 2006 to 2012 and then increased from 2012 to 2017, whereas cocaine-involved death rates without synthetic opioids increased from 2003 to 2006, decreased from 2006 to 2010, and then increased from 2010 to 2017 (Figure 1).

**FIGURE 1:**
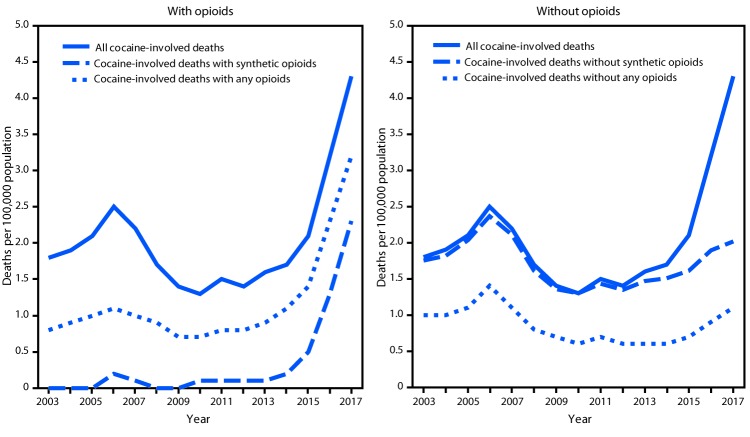
Age-adjusted rates* of drug overdose deaths^†^ involving cocaine^§^ with and without synthetic opioids other than methadone (synthetic opioids) and any opioids^¶^ — United States, 2003–2017**^,^^††^ **Source:** National Vital Statistics System, Mortality File. https://wonder.cdc.gov/. * Rate per 100,000 population age-adjusted to the 2000 U.S. standard population using the vintage year population of the data year. ^†^ Deaths are classified using the *International Classification of Diseases, Tenth Revision* (ICD-10). Drug overdoses are identified using underlying cause-of-death codes X40–X44 (unintentional), X60–X64 (suicide), X85 (homicide), and Y10–Y14 (undetermined). ^§^ Drug overdose deaths, as defined, that involve cocaine (T40.5). ^¶^ Drug overdose deaths, as defined, that involve any opioid (T40.0–T40.4 and T40.6) and synthetic opioids other than methadone (T40.4). ** Because deaths might involve more than one drug, some deaths are included in more than one category. In 2017, 12% of drug overdose deaths did not include information on the specific type of drug(s) involved. Some of these deaths might have involved opioids or stimulants. ^††^ Joinpoint regression examining changes in trends during 2003–2017 indicated that cocaine-involved overdose death rates remained stable from 2003 to 2006, then decreased annually by 10.8% (95% confidence interval [CI] = −18.1 to −3.0) from 2006 to 2012, followed by a 28.5% (CI = 19.8–37.9) annual increase from 2012 to 2017. Death rates involving cocaine and any opioid remained stable from 2003 to 2013, then increased annually by 41.6% (CI = 29.1–55.2) from 2013 to 2017. Death rates involving cocaine and synthetic opioids remained stable from 2003 to 2012, then increased annually by 114.2% (CI = 82.5–151.5) from 2012 to 2017. Death rates involving cocaine without any opioid remained stable from 2003 to 2006, then decreased annually by 13.8% (CI = −21.5 to −5.3) from 2006 to 2012, followed by a 14.9% (CI = 4.8–26.1) annual increase from 2012 to 2017. Death rates involving cocaine without synthetic opioids increased annually by 11.4% (CI = 2.1–21.6) from 2003 to 2006, then decreased annually by 14.9% (CI = −22.2 to −7.0) from 2006 to 2010, followed by a 6.9% annual increase (CI = 4.4–9.4) from 2010 to 2017.

In 2017, a total of 10,333 deaths involving psychostimulants occurred, representing 14.7% of drug overdose deaths and a 37.0% increase from 2016 (Table). During 2016–2017, the age-adjusted rate for psychostimulant-involved deaths increased by 33.3%. Approximately half (50.4%) of psychostimulant-involved deaths also involved opioids in 2017. Psychostimulant-involved death rates increased among both sexes and among persons aged ≥15 years, whites, blacks, non-Hispanic American Indians/Alaska Natives (AI/AN), non-Hispanic Asian/Pacific Islanders (A/PI), and Hispanics. The largest relative rate increase occurred among females aged 25–44 years (48.0%). Among racial/ethnic groups, the largest relative rate increase occurred among whites (40.0%), whereas AI/AN experienced the largest absolute rate increase (1.6 per 100,000) and the highest death rate (8.5) in 2017. Counties in medium metro areas experienced the largest absolute rate increase (1.3 per 100,000), and the largest relative rate increase (46.4%). Among Census regions, both the largest relative increase (63.2%) and the largest absolute rate increase (1.2) occurred in the Midwest, whereas the highest psychostimulant-involved death rate (5.3) occurred in the West. Death rates increased in 17 states, with the largest relative increases in Ohio (130.4%) and West Virginia (94.3%), and the largest absolute rate increases in West Virginia (6.6 per 100,000) and Kentucky (3.3). In 2017, the highest death rates were in West Virginia (13.6 per 100,000) and Alaska (9.1).

During 2003–2017, rates for all psychostimulant-involved deaths increased from 2010 to 2017. Death rates involving psychostimulants and any opioid increased from 2003 to 2010, followed by sharper increases from 2010 to 2015 and from 2015 to 2017. Death rates involving psychostimulants and synthetic opioids increased from 2010 to 2015, followed by a sharper increase from 2015 to 2017 (Figure 2). Rates of psychostimulant-involved deaths without any opioid involvement increased from 2008 to 2017, and rates without synthetic opioid involvement increased from 2008 to 2017 (Figure 2).

**FIGURE 2:**
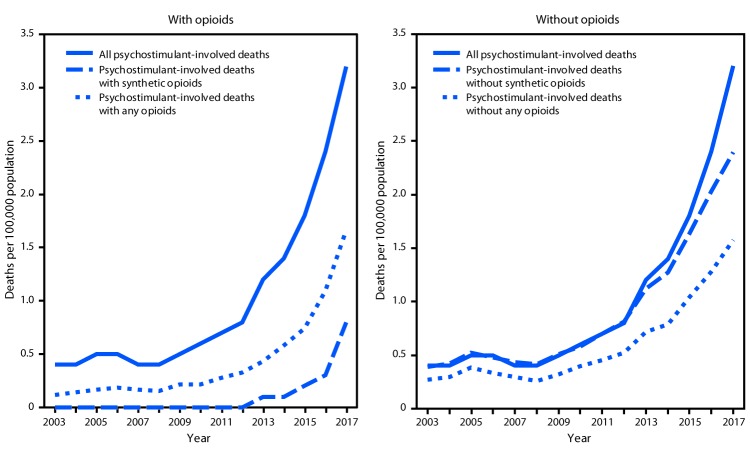
Age-adjusted rates* of drug overdose deaths^†^ involving psychostimulants with abuse potential^§^ (psychostimulants) with and without synthetic opioids other than methadone (synthetic opioids) and any opioids^¶^ — United States, 2003–2017**^,^^††^ **Source:** National Vital Statistics System, Mortality File. https://wonder.cdc.gov/. * Rate per 100,000 population age-adjusted to the 2000 U.S. standard population using the vintage year population of the data year. ^†^ Deaths are classified using the *International Classification of Diseases, Tenth Revision* (ICD-10). Drug overdoses are identified using underlying cause-of-death codes X40–X44 (unintentional), X60–X64 (suicide), X85 (homicide), and Y10–Y14 (undetermined). ^§^ Drug overdose deaths, as defined, that involve psychostimulants with abuse potential (T43.6). ^¶^ Drug overdose deaths, as defined, that involve any opioid (T40.0-T40.4, and T40.6) and synthetic opioids other than methadone (T40.4). ** Because deaths might involve more than one drug, some deaths are included in more than one category. In 2017, 12% of drug overdose deaths did not include information on the specific type of drug(s) involved. Some of these deaths may have involved opioids or stimulants. ^††^ Joinpoint regression examining changes in trends during 2003–2017 indicated that psychostimulant-involved overdose death rates remained stable from 2003 to 2010, then increased annually by 28.6% (95% confidence interval [CI] = 25.5–31.8) from 2010 to 2017. Death rates involving psychostimulants and any opioid increased annually by 6.9% (CI = 1.0–13.1) from 2003 to 2010, then increased annually by 28.2% (CI = 18.2–39.1) from 2010 to 2015, followed by a 50.8% (CI = 31.6–72.8) annual increase from 2015 to 2017. Death rates involving psychostimulants and synthetic opioids were greater than zero only during 2010–2017. From 2010 to 2015, these rates increased annually by 44.7% (CI = 2.8–103.5), followed by a 142.8% (CI = 43.7–310.2) annual increase from 2015 to 2017. Death rates involving psychostimulants without any opioids remained stable from 2003 to 2008, then increased annually by 22.3% (CI = 20.6–24.0) from 2008 to 2017. Death rates involving psychostimulants without synthetic opioids remained stable from 2003 to 2008, then increased annually by 22.3% (CI = 20.7–23.9) from 2008 to 2017.

## Discussion

Deaths involving cocaine and psychostimulants have increased in the United States in recent years; among 70,237 drug overdose deaths in 2017, nearly a third (23,139 [32.9%]) involved cocaine, psychostimulants, or both. From 2016 to 2017, death rates involving cocaine and psychostimulants each increased by approximately one third, and increases occurred across all demographic groups, Census regions, and in several states. In 2017, nearly three fourths of cocaine-involved and roughly one half of psychostimulant-involved overdose deaths, respectively, involved at least one opioid. After initially peaking in 2006, trends in overall cocaine-involved death rates declined through 2012, when they began to rise again. The 2006–2012 decrease paralleled a decline in cocaine supply coupled with an increase in cost.^¶¶¶^ Similar patterns in death rates involving both cocaine and opioids were observed, with increases for cocaine- and synthetic opioid-involved deaths occurring from 2012 to 2017. From 2010 to 2017, increasing rates of deaths involving psychostimulants occurred and persisted even in the absence of opioids. Drug overdoses continue to evolve along with emerging threats, changes in the drug supply, mixing of substances with or without the user’s knowledge, and polysubstance use (*3*–*8*). In addition, the availability of psychostimulants, particularly methamphetamine, appears to be increasing across most regions.**** In 2017, among drug products obtained by law enforcement that were submitted for laboratory testing, methamphetamine and cocaine were the most and third most frequently identified drugs, respectively.^††††^ Previous studies also found that heroin and synthetic opioids (e.g., illicitly-manufactured fentanyl) have contributed to increases in stimulant-involved deaths (*3*,*9*,*10*). Current findings further support that increases in stimulant-involved deaths are part of a growing polysubstance landscape. Although synthetic opioids appear to be driving much of the increase in cocaine-involved deaths, increases in psychostimulant-involved deaths have occurred largely without opioid co-involvement; however, recent data suggest increasing synthetic opioid involvement in these deaths.

The findings in this report are subject to at least four limitations. First, at autopsy, substances tested for and circumstances under which tests are performed vary by time and jurisdiction. Therefore, recent improvements in toxicologic testing might account for some reported increases. Second, 15% and 12% of death certificates in 2016 and 2017, respectively, did not include mention of specific drugs involved. The percentage of death certificates with at least one drug specified varied widely by state, ranging from 54.7% to 99.3% in 2017, limiting comparisons across states. Third, potential racial misclassification might lead to underestimates for certain groups, primarily AI/AN and A/PI.^§§§§^ Finally, certain trend analyses were limited, given small numbers of deaths and the inability to calculate stable rates among some stimulant-opioid drug combinations before 2003.

Preliminary 2018 data indicate continued increases in drug overdose deaths.^¶¶¶¶^ The rise in deaths involving cocaine and psychostimulants and the continuing evolution of the drug landscape indicate a need for a rapid, multifaceted, and broad approach that includes more timely and comprehensive surveillance efforts to inform tailored and effective prevention and response strategies. CDC currently funds 45 states and DC for opioid surveillance***** and/or prevention activities.^†††††^ The contribution of opioids to increases in stimulant-involved overdose deaths underscores the importance of continued opioid overdose surveillance and prevention measures, including existing efforts to expand naloxone availability to persons at risk for drug overdose. CDC is expanding drug overdose surveillance efforts to include stimulants and is implementing multiple, evidence-based opioid prevention efforts, such as enhancing linkage to care, building state and local capacity, and public health/public safety collaborations.^§§§§§^ Because some stimulant deaths are also increasing without opioid co-involvement, prevention and response strategies need to evolve accordingly. Increased efforts are required to identify and improve access to care for persons using stimulants, implement upstream prevention efforts focusing on shared risk and protective factors that address substance use/misuse, and improve risk reduction messaging (e.g., not using alone). Continued collaborations among public health, public safety, and community partners are critical to understanding the local illicit drug supply and reducing risk as well as linking persons to medication-assisted treatment and risk-reduction services.

**Summary**

**What is already known about this topic?**

Overdose deaths involving cocaine and psychostimulants continue to increase. During 2015–2016, age-adjusted cocaine-involved and psychostimulant-involved death rates increased by 52.4% and 33.3%, respectively.

**What is added by this report?**

From 2016 to 2017, death rates involving cocaine and psychostimulants increased across age groups, racial/ethnic groups, county urbanization levels, and multiple states. Death rates involving cocaine and psychostimulants, with and without opioids, have increased. Synthetic opioids appear to be the primary driver of cocaine-involved death rate increases, and recent data point to increasing synthetic opioid involvement in psychostimulant-involved deaths.

**What are the implications for public health practice?**

Continued increases in stimulant-involved deaths require expanded surveillance and comprehensive, evidence-based public health and public safety interventions.

## Footnotes

*
https://wonder.cdc.gov .

†
https://www.cdc.gov/nchs/nvss/vsrr/drug-overdose-data.htm .

§
https://www.cdc.gov/nchs/nvss/mortality_public_use_data.htm .

¶ T40.0 (opium), T40.1 (heroin), T40.2 (natural/semisynthetic opioids), T40.3 (methadone), T40.4 (synthetic opioids other than methadone), and T40.6 (other and unspecified narcotics).

** A death involving both cocaine and psychostimulants with abuse potential (e.g., methamphetamine) would be included in both the cocaine and the psychostimulant with abuse potential death rates.

†† Age-adjusted death rates were calculated by applying age-specific death rates to the 2000 U.S. Census standard population age distribution https://www.cdc.gov/nchs/data/nvsr/nvsr61/nvsr61_04.pdf.

§§ Categories of 2013 NCHS Urban-Rural Classification Scheme for Counties (https://www.cdc.gov/nchs/data_access/urban_rural.htm): *Large central metro:* Counties in metropolitan statistical areas (MSAs) of ≥1 million population that 1) contain the entire population of largest principal city of the MSA, or 2) have their entire population contained in the largest principal city of the MSA, or 3) contain at least 250,000 inhabitants of any principal city of the MSA; *Large fringe metro:* Counties in MSAs of ≥1 million population that did not qualify as large central metro counties; *Medium metro:* Counties in MSAs of populations of 250,000–999,999; *Small metro:* Counties in MSAs of populations less than 250,000; *Micropolitan (nonmetropolitan counties):* counties in micropolitan statistical areas; *Noncore (nonmetropolitan counties):* nonmetropolitan counties that did not qualify as micropolitan.

¶¶ *Northeast:* Connecticut, Maine, Massachusetts, New Hampshire, New Jersey, New York, Pennsylvania, Rhode Island, and Vermont; *Midwest:* Illinois, Indiana, Iowa, Kansas, Michigan, Minnesota, Missouri, Nebraska, North Dakota, Ohio, South Dakota, and Wisconsin; *South:* Alabama, Arkansas, Delaware, District of Columbia, Florida, Georgia, Kentucky, Louisiana, Maryland, Mississippi, North Carolina, Oklahoma, South Carolina, Tennessee, Texas, Virginia, and West Virginia; *West:* Alaska, Arizona, California, Colorado, Hawaii, Idaho, Montana, Nevada, New Mexico, Oregon, Utah, Washington, and Wyoming.

*** State-level analyses comparing death rates from 2016 to 2017 included 34 states and DC that met the following criteria: 1) >80% of drug overdose death certificates named at least one specific drug in 2016 and 2017; 2) change from 2016 to 2017 in the percentage of death certificates reporting at least one specific drug was <10 percentage points; and 3) ≥20 deaths occurred during 2016 and 2017 in at least one drug category examined. States whose reporting of any specific drug or drugs involved in an overdose changed by ≥10 percentage points from 2016 to 2017 were excluded because drug-specific overdose numbers and rates might have changed substantially from 2016 to 2017 as a result of changes in reporting.

††† Z-tests were used if the number of deaths was ≥100, and a p-value of <0.05 was considered to be statistically significant. Nonoverlapping confidence intervals based on the gamma method were used if the number of deaths was <100 in 2015 or 2016. Note that the method of comparing confidence intervals is a conservative method for statistical significance; caution should be observed when interpreting a nonsignificant difference when the lower and upper limits being compared overlap only slightly. https://www.cdc.gov/nchs/data/NVSR/NVSR61/NVSR61_04.pdf.

§§§ For all analyses, a p-value of <0.05 was considered to be statistically significant. https://surveillance.cancer.gov/joinpoint/.

¶¶¶
https://www.justice.gov/archive/ndic/pubs38/38661/cocaine.htm .

****
https://www.dea.gov/sites/default/files/2018-11/DIR-032-18%202018%20NDTA%20final%20low%20resolution.pdf .

††††
https://www.nflis.deadiversion.usdoj.gov/DesktopModules/ReportDownloads/Reports/NFLIS-Drug-AR2017.pdf .

§§§§
https://www.cdc.gov/nchs/data/series/sr_02/sr02_172.pdf .

¶¶¶¶
https://www.cdc.gov/nchs/nvss/vsrr/drug-overdose-data.htm .

*****
https://www.cdc.gov/drugoverdose/foa/state-opioid-mm.html .

††††† https://www.cdc.gov/drugoverdose/states/state_prevention.html; https://www.cdc.gov/drugoverdose/foa/ddpi.html.

§§§§§
https://www.cdc.gov/drugoverdose/od2a/index.html .
